# Linear relationship between percentage-based waist-to-height ratio and the risk of gallstones among US adults: A cross-sectional study from NHANES 2017 to 2020

**DOI:** 10.1097/MD.0000000000047151

**Published:** 2026-01-09

**Authors:** Shuangshuang Hou, Shenlin Ji, Yanan Huang, He Li, Jin Huang, Yaoyuan Chang, Jiajun Yin, Zhequn Nie, Ju Wu

**Affiliations:** a Department of General Surgery, Fuyang Normal University Second Affiliated Hospital, Fuyang, Anhui Province, China; b Department of Graduate School, Dalian Medical University, Dalian, Liaoning Province, China; c Department of General Surgery, Affiliated Zhongshan Hospital of Dalian University, Dalian, Liaoning Province, China.

**Keywords:** cross-sectional study, gallstones, waist-to-height ratio, NHANES, obesity

## Abstract

The influence of obesity on the formation of gallstones has been extensively studied. The waist-to-height ratio (WHtR) is an effective tool for assessing body fat and obesity levels. This study aimed to investigate the linear correlation between percentage-based WHtR and gallstone prevalence. Data from 7731 participants in the National Health and Nutrition Examination Survey from 2017 to 2020 were used in this cross-sectional study. Weighted logistic regression, trend tests, restricted cubic splines (RCS), subgroup and interaction analyses were employed to evaluate the association between WHtR and gallstones. In the fully adjusted logistic regression model, each 1% increase in WHtR was associated with a 4.3% increase in the risk of gallstones (odds ratio = 1.043, 95% confidence interval: 1.034–1.052). Compared to the lowest quartile of WHtR, the highest quartile had a 201.5% increased risk of gallstones (odds ratio = 3.015, 95% confidence interval: 2.239–4.061), demonstrating a dose–response relationship (*P* for trend < .01). RCS indicate a positive linear relationship between WHtR and the odds of gallstones (*P* for nonlinear = .297). The association between WHtR and gallstone risk persisted in subgroup analyses. Interaction analyses showed that age and gender have an interactive effect on the association. There was a positive linear relationship between WHtR based on percentage and the prevalence of gallstones, displaying a dose–response relationship. Maintaining an appropriate WHtR may reduce the risk of gallstones.

## 1. Introduction

Gallstone disease ranks among the most frequent digestive disorders and constitutes a major global public health problem. The incidence of gallstones varies considerably across different geographic regions and ethnic groups. It is estimated that approximately 20 million people in the United States are affected by gallstone disease, with an overall incidence rate in Europe of 18.8%,^[1]^ and relatively lower rates in Asia.^[2]^ In recent years, due to changes in lifestyle and dietary habits, the incidence of gallstones has been gradually increasing. Gallstones are a significant risk factor for biliary cancers, especially gallbladder cancer.^[3]^ Most patients with gallstones typically do not exhibit obvious clinical symptoms. However, a minority may experience various severe complications, potentially life-threatening.^[4]^ Several factors, such as age, gender, genetics, inactivity, and elements of metabolic syndrome like central obesity, dyslipidemia, and type 2 diabetes, affect the development of gallstones.^[5-8]^ However, simple and reliable predictors for gallstone risk are currently unavailable.

In recent years, obesity and its associated chronic diseases have significantly increased, posing a serious threat to global public health and safety. Statistics indicate that currently over 40% of American adults are affected by obesity, and this proportion is projected to rise to 48.9% by 2030, with >1 billion adults worldwide expected to be obese.^[9]^ Additionally, research shows that obesity is an independent risk factor for metabolic syndrome and gallstones,^[10]^ with the link between obesity and gallstones receiving increasing attention as this risk factor is mostly modifiable and controllable.^[11]^ In clinical practice, the indices used to evaluate obesity primarily include body mass index (BMI), waist circumference (WC), waist-to-hip ratio (WHR), and body weight.^[12]^ BMI is one of the most widely used indices for assessing obesity, but it does not accurately assess fat and its distribution (including visceral fat), particularly as BMI does not consider muscle mass, nor can it precisely determine the accumulation of abdominal or visceral fat, and is easily influenced by age, gender, and ethnicity.^[13]^ WC is highly correlated with BMI, thus it is not suitable for use as an independent obesity assessment index.^[14]^ For WHR, some scholars believe that a combined assessment with BMI is necessary for more accurate evaluation.^[15]^ Therefore, relying solely on BMI or WC is not highly reliable for assessing obesity; researchers like Ashwell have proposed that the waist-to-height ratio (WHtR) is an effective predictive indicator of abdominal fat, as WHtR reflects the accumulation of abdominal fat more accurately than WC.^[16]^ Hence, WHtR is recommended as an anthropometric measure for assessing central obesity.^[17]^ It is low-cost, easy to measure, and has a high predictive value in clinical settings. Earlier studies have found that WHtR is linked to the prevalence of gallstones.^[18]^ However, the linear relationship between the 2 has not been thoroughly investigated.

In this study, a cross-sectional analysis was conducted using the National Health and Nutrition Examination Survey (NHANES) database from 2017 to 2020, where the WHtR was converted into a percentage and its association with gallstone risk was evaluated.

## 2. Materials and methods

### 2.1. Study population

**Figure 1.**
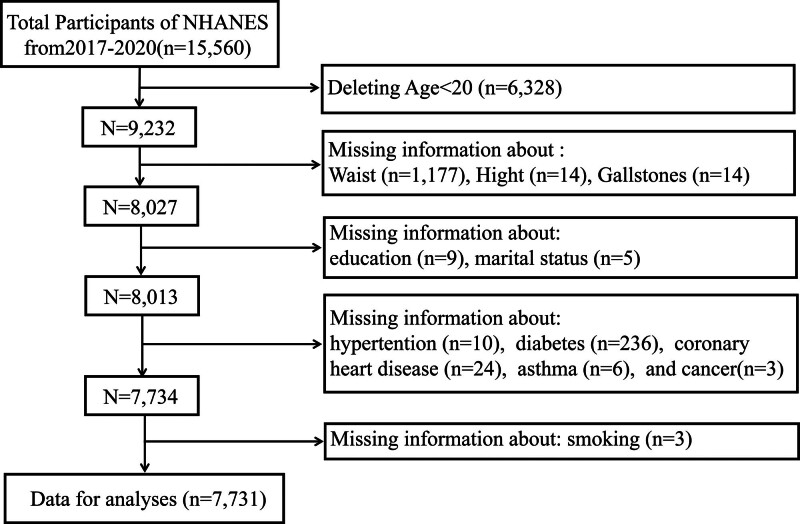
The flowchart of participant selection from NHANES 2017 to 2020 (N = 7731). NHANES = National Health and Nutritional Examination Survey.

Information from the NHANES database was used in this study, including demographic, dietary, laboratory, and questionnaire data. The analysis followed the sample weighting recommended by the official NHANES website and accounted for the complexities of the multi-stage cluster survey. Data from 15,560 participants was analyzed in the study conducted between 2017 and 2020. A total of 7731 eligible participants were included in the final analysis after excluding those under 20 years old or missing data on WC, height, gallstone history, education, marital status, smoking and the prevalence of underlying diseases (Fig. 1). Approval from the National Center for Health Statistics Ethical Review Board was granted to each participant, who also signed a written informed consent form. Consequently, further ethical review was unnecessary.^[19]^

### 2.2. Exposure and outcome variables

The exposure variable In this study is defined as the percentage WHtR, calculated using the formula: WHtR = (WC [cm]/Height [cm]) × 100%. The outcome variable is whether participants have gallstones, assessed through the questionnaire item mcq550 – “Has a doctor or other healthcare professional ever diagnosed you with gallstones?.” Those who responded “yes” were identified as having gallstones. This practical method has been extensively utilized in prior research.^[20,21]^

### 2.3. Covariates

This study included several covariates potentially affecting the relationship between the WHtR and gallstones. The covariates include age (years), gender, race, educational level (less than high school, high school, more than school), marital status (live with partner or live alone), poverty income ratio (PIR: 0–1.5, 1.5–3.5, >3.5), physical activity levels, total cholesterol (TC; mg/dL), smoking status (Has smoked a minimum of 100 cigarettes in their lifetime?), alcohol consumption (based on questionnaire alq121: How frequently did you consume alcoholic beverages over the last year? Responses of “1–2 times” or more frequently were considered indicative of drinking), and physical activity status (classified as mild, moderate, vigorous, or unclear based on the number of days engaging in moderate-intensity activities according to questionnaire paq670). The study also accounted for the presence of hypertension, diabetes, coronary heart disease, asthma, and cancer (participants answering “yes” on the medical conditions questionnaire were considered as having these diseases). Moreover, since insulin resistance is a risk factor for gallstone development,^[22]^ additional data on glycemia and BMI were collected, and the metabolic score for insulin resistance (METS-IR) was calculated: METS-IR = = Ln[2 × glycemia (mg/dL) + TC (mg/dL)] × BMI/Ln[high-density lipoprotein cholesterol (mg/dL)]. An automatic biochemistry analyzer was used to measure all biochemical indices following a fast of over 8 hours.

### 2.4. Handling of missing values

Two strategies were used in this study to manage missing covariate values. We transformed continuous variables with significant missing data into categorical variables, placing participants with missing information into an “unclear” category.^[23]^ For variables with fewer missing values, missing data were handled using multiple imputation via the MICE package in R.^[24,25]^ The NHANES website provides free access to detailed measurement methods for the variables used in this study.

### 2.5. Statistical analysis

Normally distributed continuous variables were represented by mean ± standard deviation and evaluated using chi-square or *t*-tests for various covariates.^[26]^ Weighted logistic regression was used to analyze the relationship between WHtR and the risk of gallstones. Three models were developed in this analysis: model 1 was unadjusted; model 2 adjusted for age, gender, and race; and model 3 adjusted for all covariates. WHtR was categorized into quartiles for trend analysis. Restricted cubic splines (RCS) were used to explore potential nonlinear associations between WHtR and gallstone risk.^[27]^ Interaction tests assessed the stability of this relationship across different subgroups.^[28]^ Statistical analyses were performed using Empower (version 5.0; X&Y Solutions Inc., Boston) and R (version 4.2.0; R Foundation, Vienna, Austria), with a *P*-value of <.05 considered statistically significant in all analyses.

## 3. Results

### 3.1. Baseline characteristics

This study included a total of 7731 participants, of whom 51.3% were female and 48.7% were male (Table 1). The average age and WHtR of participants were 50.3 ± 17.4 years and 60.51 ± 10.26, respectively, with a 10.4% prevalence of gallstones. Participants with gallstones were generally older, had a higher WHtR, were predominantly non-Hispanic White, and were more likely to be drinkers and smokers compared to those without gallstones. They also exhibited a higher prevalence of comorbidities (*P* < .05). However, the comparison between the 2 groups revealed no significant differences in TC levels, educational level, and marital status (*P* > .05).

**Table 1:** Distribution of selected characteristics of the US adults from NHANES 2017 to 2020 (n = 7731).

Characteristics	Total	Non-gallstones	Gallstones	*P*-value
	N = 7731	N = 6929 (89.6%)	N = 802 (10.4%)	
Age (yr)	50.3 ± 17.4	49.4 ± 17.4	57.7 ± 15.8	<.001
WHtR (%)	60.51 ± 10.26	59.86 ± 10.04	66.18 ± 10.44	<.001
Gender, n (%)				<.001
Male	3765 (48.7)	3535 (51.0)	230 (28.7)	
Female	3966 (51.3)	3394 (49.0)	572 (71.3)	
Race, n (%)				<.001
Mexican American	904 (11.7)	798 (11.5)	106 (13.2)	
Other Hispanic	782 (10.1)	689 (9.9)	93 (11.6)	
Non-Hispanic White	2705 (35.0)	2369 (34.2)	336 (41.9)	
Non-Hispanic Black	2055 (26.6)	1890 (27.3)	165 (20.6)	
Other race	1285 (16.6)	1183 (17.1)	102 (12.7)	
Educational level, n (%)				.419
Less than high school	1404 (18.2)	1267 (18.3)	137 (17.1)	
High school	1881 (24.3)	1672 (24.1)	209 (26.0)	
More than high school	4446 (57.5)	3990 (57.6)	456 (56.9)	
Marital status, n (%)				.528
Live with partner	4508 (58.3)	4032 (58.2)	476 (59.4)	
Live alone	3223 (41.7)	2897 (41.8)	326 (40.6)	
PIR, n (%)				.047
<1.3	1832 (23.7)	1647 (23.8)	185 (23.1)	
1.3–3.5	2634 (34.1)	2327 (33.6)	307 (38.3)	
≥3.5	2197 (28.4)	1983 (28.6)	214 (26.7)	
Unclear	1068 (13.8)	972 (14.0)	96 (11.9)	
Drinking, n (%)				<.001
Yes	5284 (68.4)	4799 (69.3)	485 (60.5)	
No	2447 (31.6)	2130 (30.7)	317 (39.5)	
Smoked, n (%)				.003
Yes	3225 (41.7)	2851 (41.2)	374 (46.6)	
No	4506 (58.3)	4078 (58.8)	428 (53.4)	
TC, n (%)				.606
<185.99 mg/dL	3827 (49.5)	3417 (49.3)	410 (51.1)	
≥185.99 mg/dL	3402 (44.0)	3062 (44.2)	340 (42.4)	
Unclear	502 (6.5)	450 (6.5)	52 (6.5)	
METS-IR, n (%)				<.001
<44.2	2031 (26.3)	1875 (27.1)	156 (19.5)	
≥44.2	1561 (20.2)	1337 (19.3)	224 (27.9)	
Unclear	4139 (53.5)	3717 (53.6)	422 (52.6)	
Hypertension, n (%)				<.001
Yes	2897 (37.5)	2461 (35.5)	436 (54.4)	
No	4834 (62.5)	4468 (64.5)	366 (45.6)	
Diabetes, n (%)				<.001
Yes	1181 (15.3)	971 (14.0)	210 (26.2)	
No	6550 (84.7)	5958 (86.0)	592 (73.8)	
Asthma, n (%)				<.001
Yes	1223 (15.8)	1062 (15.3)	161 (20.1)	
No	6508 (84.2)	5867 (84.7)	641 (79.9)	
Heart disease, n (%)				<.001
Yes	330 (4.3)	259 (3.7)	71 (8.8)	
No	7401 (95.7)	6670 (96.3)	731 (91.2)	
Cancer, n (%)				<.001
Yes	781 (10.1)	639 (9.2)	142 (17.7)	
No	6950 (89.9)	6290 (90.8)	660 (82.3)	
Activity, n (%)				<.001
Mild	1039 (13.4)	958 (13.8)	81 (10.1)	
Moderate	1708 (22.1)	1538 (22.2)	170 (21.2)	
Vigorous	450 (5.8)	416 (6.0)	34 (4.2)	
Unclear	4534 (58.7)	4017 (58.0)	517 (64.5)	

Mean ± SD for continuous variables: the *P*-value was calculated by the weighted linear regression model; (%) for categorical variables: the *P*-value was calculated by the weighted chi-square test.

METS-IR = metabolic score for insulin resistance, NHANES = National Health and Nutritional Examination Survey, PIR = poverty income ratio, SD = standard deviation, TC = total cholesterol, WHtR = waist-to-height ratio.

### 3.2. Relationship between WHtR and gallstones

**Figure 2.**
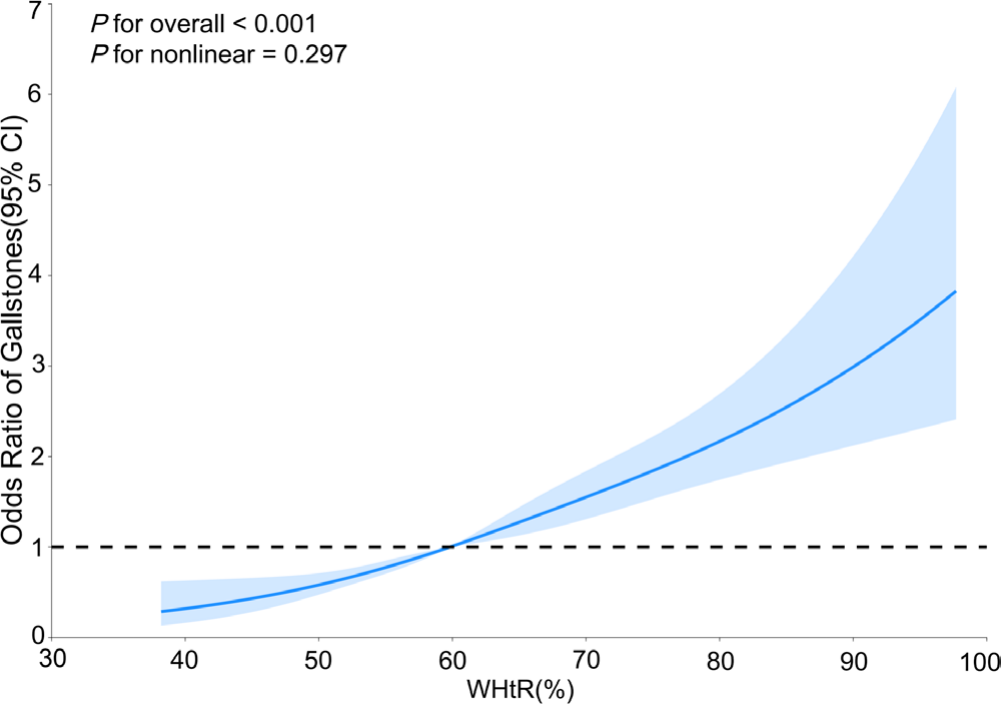
Restricted cubic spline analysis of WHtR and the risk of gallstones in US adults from NHANES 2017 to 2020 (N = 7731). CI = confidence interval, NHANES = National Health and Nutritional Examination Survey, WHtR = waist to height ratio.

Weighted logistic regression analysis indicated that increased WHtR was associated with a higher risk of gallstones across differently adjusted models (Table 2). In the model that adjusted for all covariates, this association remained significantly stable (odds ratio = 1.043; 95% confidence interval: 1.034–1.052; *P* < .01). When WHtR was split into quartiles and fully adjusted, the risk of gallstones in the highest quartile was higher than in the lowest quartile (odds ratio = 3.015; 95% confidence interval: 2.239–4.061; *P* < .01). Additionally, trend tests were significantly positive in each of the 3 models (*P* for trend* *< 0.01). RCS demonstrated a linear positive correlation between WHtR and the risk of gallstones (*P* for nonlinear = 0.297; Fig. 2).

**Table 2:** Logistic regression analysis on the association between WHtR and gallstones in the US adults from NHANES 2017 to 2020 (n = 7731).

Exposure	Model 1 (OR [95% CI])	Model 2 (OR [95% CI])	Model 3 (OR [95% CI])
WHtR (continuous, %)	1.06 (1.05, 1.07)	1.05 (1.04, 1.06)	1.04 (1.03, 1.05)
WHtR (quartiles, %)			
Q1 (≤53.2)	1.00	1.00	1.00
Q2 (53.3–59.7)	1.73 (1.30,2.31)	1.39 (1.04,1.86)	1.37 (1.02, 1.84)
Q3 (59.8–66.8)	3.33 (2.56,4.33)	2.43 (1.85,3.19)	2.23 (1.68,2.98)
Q4 (≥66.9)	5.24 (4.06,6.75)	3.64 (2.79,4.74)	3.02 (2.24,4.06)
*P* for trend	<.01	<.01	<.01

Model 1: Unadjusted.

Model 2: Adjusted for age, sex, and race.

Model 3: Adjusted for age, sex, race, marital status, education level, alcohol consumption, smoked, physical activity, diabetes, hypertension, heart disease, asthma, cancer, TC, PIR, and METS-IR.

CI = confidence interval, METS-IR = metabolic score for insulin resistance, NHANES = National Health and Nutritional Examination Survey, OR = odds ratio, PIR = poverty income ratio, Q1 = 1st quartile, Q2 = 2nd quartile, Q3 = 3rd quartile, Q4 = 4th quartile, TC = total cholesterol, WHtR = waist-to-height ratio.

### 3.3. Subgroup and interaction analysis

**Figure 3.**
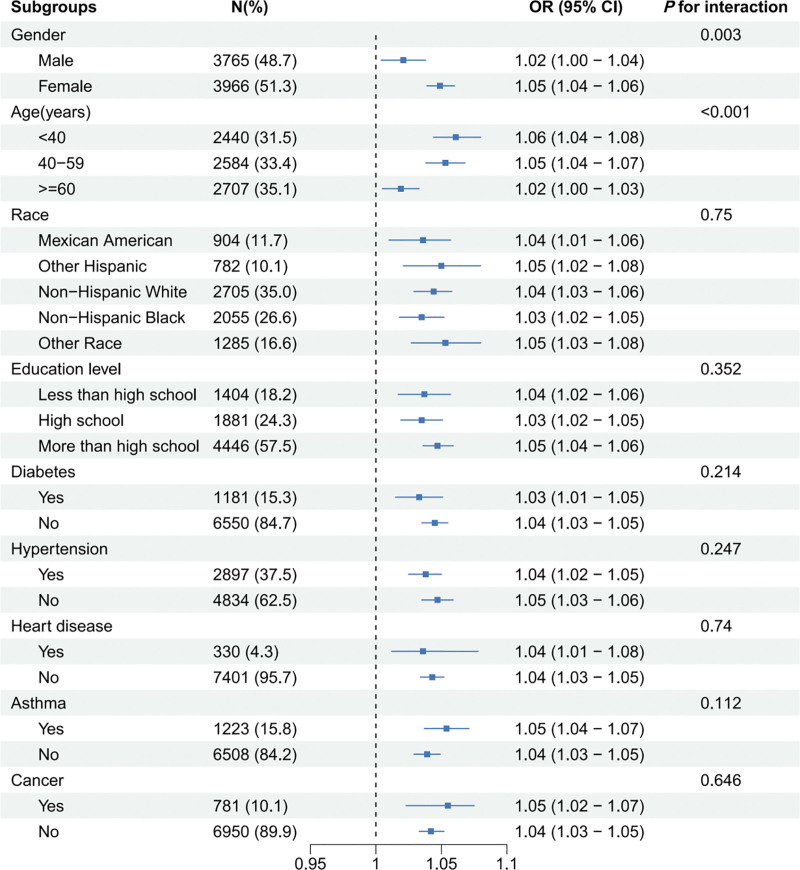
Subgroup and interaction analysis of the association between WHtR and the risk of gallstones in US adults from NHANES 2017 to 2020 (N = 7731). CI = confidence interval, NHANES = National Health and Nutritional Examination Survey, OR = odds ratio, WHtR = waist to height ratio.

To assess the consistency of the relationship between WHtR and gallstone risk, we conducted subgroup and interaction analyses and explored potential population differences. As shown in Figure 3, the association between WHtR and the risk of gallstones was significant across all subgroups, particularly notable in women under the age of 60. Interaction analysis revealed that age and gender have interactive effects on the relationship (*P* < .05). However, there were no significant interactive effects of race, diabetes, hypertension, heart disease, asthma, or cancer on the relationship between WHtR and gallstones (*P* > .05).

### 3.4. Sensitivity analysis

To further assess robustness, we conducted a sensitivity analysis by excluding participants with hypertension, diabetes, coronary heart disease, asthma, and cancer, and repeated the RCS analysis. After adjusting for all covariates, the analysis showed that the association between WHtR and gallstone risk remained consistent with the primary analysis (*P* for overall < .001, *P* for nonlinear = .276, Fig. S1, Supplemental Digital Content, https://links.lww.com/MD/R130).

## 4. Discussion

In this cross-sectional study involving 7731 US adults, we found a correlation between higher WHtR and increased risk of gallstones, which remained stable even after adjusting for all the covariates. The trend test reveals a dose–response connection between WHtR and the odds of gallstones. A linear relationship is shown by the RCS results, which are consistent with the findings from logistic regression. This suggests that a higher WHtR may elevate the risk of developing gallstones. Subgroup analysis revealed that this correlation remains consistent across different demographics, particularly pronounced in women under 60, with age and gender having an interactive effect on the relationship. These results highlight the possible significance of WHtR in evaluating and controlling the risk of gallstones.

There have been no studies so far that specifically address the linear relationship between WHtR and gallstones, with prior research focusing on other obesity metrics linked to gallstones.^[29,30]^ Chou et al highlighted that WHtR is an effective predictor for gallstones in a cross-sectional study involving 2263 Taiwan individuals.^[31]^ Traditional obesity measures like BMI and WC are also widely regarded as risk factors for gallstones and gallbladder polyps. For instance, research from Denmark involving 77,679 people revealed that a rise in BMI is an independent risk factor for symptomatic gallstone disease.^[32]^ Ke et al reported a positive correlation between waist-weight index and the prevalence of gallstones.^[23]^ Whereas, a joint study based on databases from the UK and Finland demonstrated independent causal relationships between BMI and WC with gallstones.^[33]^ Barahona Ponce et al also found a causal link between BMI and gallstone risk in European populations.^[34]^ Concerning the impact of gender and age on gallstones, research by Amir Reza Radmard suggests that different genders prefer different anthropometric indices to assess gallstone risk, with WHR being the preferred metric among men.^[35]^ Nevertheless, the majority of research indicates that women are more prone to gallstones than men.^[6,36]^ A nationwide study from China indicated that the prevalence of gallstones significantly increases with age.^[37]^ Overall, our study further corroborates the significant correlation between higher WHtR and increased gallstone disease risk. This points to the possibility that an elevated WHtR plays a major role in gallstone development. However, further investigation is necessary to understand the biological mechanisms and clinical outcomes related to this connection.

There are several possible mechanisms that could account for the positive relationship between WHtR and gallstones. First, in obesity, metabolic changes occur in different organs, such as the liver and gallbladder, characterized by the liver’s excessive bile secretion, high blood lipid levels, and reduced intestinal movement.^[38]^ Second, obesity increases insulin resistance, leading to lipid metabolism disorders and subsequently higher gallstone incidence.^[39,40]^ Third, the activity of HMG-CoA reductase is stimulated by elevated insulin levels in the plasma, leading to an oversecretion of cholesterol.^[41]^ Additionally, the higher incidence of gallstones in females may be influenced by elevated levels of estrogen, leading to an increase in both the secretion and synthesis of cholesterol by the liver, promoting the formation of crystalline stones, thereby making the condition more common in women.^[5]^

## 5. Strengths and limitations of this study

First, our study draws from the NHANES database, recognized for its large sample size and credible data. Second, this study is the first to investigate the linear correlation between WHtR based on percentage and the risk of gallstones by adjusting for multiple confounding factors and conducting subgroup analyses. However, as biases in cross-sectional studies are inevitable, we cannot determine the temporal relationship between gallstones and WHtR. Future studies should further explore the underlying mechanisms between the 2 and develop corresponding clinical tools to predict the risk of gallstones. The study findings should also be interpreted with caution, as the main outcome measures were based on self-reported data, and the possibility of classification bias cannot be excluded. Additionally, although we considered confounders in our multivariable regression model, this relationship should still be studied prospectively. Lastly, due to the lack of data, many subjects were excluded; further studies should concentrate on collecting more complete data.

## 6. Conclusion

Our research findings suggest a positive linear relationship between the percentage-based WHtR and the prevalence of gallstones, demonstrating a dose–response relationship. Maintaining an appropriate WHtR may reduce the risk of gallstones, providing new insights for their prevention and treatment in the future.

## Acknowledgments

We express our gratitude to the NHANES database for offering their platforms and to the contributors for sharing their valuable datasets. We also appreciate all the participants involved in our current study.

## Author contributions

**Conceptualization:** Shuangshuang Hou, Shenlin Ji.

**Data curation:** Shuangshuang Hou.

**Formal analysis:** Yanan Huang.

**Investigation:** He Li, Yaoyuan Chang.

**Methodology:** Yanan Huang.

**Project administration:** Jiajun Yin, Zhequn Nie.

**Resources:** Ju Wu.

**Software:** He Li, Yaoyuan Chang.

**Supervision:** Jiajun Yin, Zhequn Nie.

**Validation:** Shuangshuang Hou, Shenlin Ji, Jin Huang.

**Visualization:** Ju Wu.

**Writing – original draft:** Shuangshuang Hou.

**Writing – review & editing:** Shenlin Ji, Jin Huang.

## Supplementary Material



## References

[1] Hao JY Zhang YP Huang XJ. Design and development of a disposable superfine catheter for visual examination of bile ducts and related animal experiments. Front Surg. 2022;9:877040. 35586506 10.3389/fsurg.2022.877040 PMC9108420

[2] Huang L Ding C Si X. Changes in the interstitial cells of Cajal in the gallbladder of guinea pigs fed a lithogenic diet. Exp Ther Med. 2021;22:823. 34131446 10.3892/etm.2021.10255 PMC8193206

[3] Park SK Andreotti G Rashid A. Polymorphisms of estrogen receptors and risk of biliary tract cancers and gallstones: a population-based study in Shanghai, China. Carcinogenesis. 2010;31:842–6. 20172949 10.1093/carcin/bgq038 PMC2864412

[4] Meng Y Meng K Zhao X. Protective effects of yinchenhao decoction on cholesterol gallstone in mice fed a lithogenic diet by regulating LXR, CYP7A1, CYP7B1, and HMGCR Pathways. Evid Based Complement Alternat Med. 2018;2018:8134918. 30310412 10.1155/2018/8134918 PMC6166389

[5] Lammert F Gurusamy K Ko CW. Gallstones. Nat Rev Dis Primers. 2016;2:16024. 27121416 10.1038/nrdp.2016.24

[6] Méndez-Sánchez N Chavez-Tapia NC Motola-Kuba D. Metabolic syndrome as a risk factor for gallstone disease. World J Gastroenterol. 2005;11:1653–7. 15786544 10.3748/wjg.v11.i11.1653 PMC4305948

[7] Chen L Yang H Li H He C Yang L Lv G. Insights into modifiable risk factors of cholelithiasis: a Mendelian randomization study. Hepatology. 2022;75:785–96. 34624136 10.1002/hep.32183 PMC9300195

[8] Weikert C Weikert S Schulze MB. Presence of gallstones or kidney stones and risk of type 2 diabetes. Am J Epidemiol. 2010;171:447–54. 20089496 10.1093/aje/kwp411

[9] Wibmer AG Becher T Eljalby M. Brown adipose tissue is associated with healthier body fat distribution and metabolic benefits independent of regional adiposity. Cell Rep Med. 2021;2:100332. 34337558 10.1016/j.xcrm.2021.100332 PMC8324464

[10] Lokhov PG Balashova EE Trifonova OP Maslov DL Ponomarenko EA Archakov AI. Mass spectrometry-based metabolomics analysis of obese patients’ blood plasma. Int J Mol Sci. 2020;21:568. 31952343 10.3390/ijms21020568 PMC7014187

[11] Ahmed MH. Ezetimbe as potential treatment for cholesterol gallstones: the need for clinical trials. World J Gastroenterol. 2010;16:1555–7. 20355232 10.3748/wjg.v16.i13.1555 PMC2848362

[12] Fan YJ Feng YJ Meng Y Su ZZ Wang PX. The relationship between anthropometric indicators and health-related quality of life in a community-based adult population: a cross-sectional study in Southern China. Front Public Health. 2022;10:955615. 36249240 10.3389/fpubh.2022.955615 PMC9554305

[13] Abboud M Haidar S Mahboub N Papandreou D Rizk R. Abdominal volume index, waist-to-height ratio, and waist circumference are optimal predictors of cardiometabolic abnormalities in a sample of Lebanese adults: a cross-sectional study. PLOS Glob Public Health. 2023;3:e0002726. 38127883 10.1371/journal.pgph.0002726 PMC10734963

[14] Clark AL Fonarow GC Horwich TB. Waist circumference, body mass index, and survival in systolic heart failure: the obesity paradox revisited. J Card Fail. 2011;17:374–80. 21549293 10.1016/j.cardfail.2011.01.009

[15] Haufs MG Zöllner YF. Waist-hip ratio more appropriate than Body Mass Index. Dtsch Arztebl Int. 2020;117:659. 10.3238/arztebl.2020.0659a PMC7829451 33357347

[16] Ashwell M Cole TJ Dixon AK. Ratio of waist circumference to height is strong predictor of intra-abdominal fat. BMJ. 1996;313:559–60. 10.1136/bmj.313.7056.559d PMC2351911 8790002

[17] Fan H Zhu Q Medrano-Gracia P Zhang X. Comparison of child adiposity indices in prediction of hypertension in early adulthood. J Clin Hypertens (Greenwich). 2019;21:1858–62. 31742895 10.1111/jch.13734 PMC8030433

[18] Zhang J Liang D Xu L. Associations between novel anthropometric indices and the prevalence of gallstones among 6,848 adults: a cross-sectional study. Front Nutr. 2024;11:1428488. 39104753 10.3389/fnut.2024.1428488 PMC11298442

[19] Liu CF Chien LW. Triglyceride glucose index and poor sleep patterns in non-diabetic adults: evidence from NHANES 2005-2016. Front Nutr. 2023;10:1051667. 36793924 10.3389/fnut.2023.1051667 PMC9922746

[20] Wang J Sun YX Xiang S. The association between blood heavy metals and gallstones: a cross-sectional study. Sci Total Environ. 2023;904:166735. 37659556 10.1016/j.scitotenv.2023.166735

[21] Zhang H Xu C Zhu X. Associations between temporal eating patterns and energy distribution patterns with gallstones: a cross-sectional study based on NHANES 2017-2018. BMC Public Health. 2024;24:2994. 39472867 10.1186/s12889-024-20512-x PMC11523901

[22] Wang J Yang J Chen Y Rui J Xu M Chen M. Association of METS-IR index with prevalence of gallbladder stones and the age at the first gallbladder stone surgery in US adults: a cross-sectional study. Front Endocrinol (Lausanne). 2022;13:1025854. 36263324 10.3389/fendo.2022.1025854 PMC9574223

[23] Ke B Sun Y Dai X Gui Y Chen S. Relationship between weight-adjusted waist circumference index and prevalence of gallstones in U.S. adults: a study based on the NHANES 2017-2020. Front Endocrinol (Lausanne). 2023;14:1276465. 37964952 10.3389/fendo.2023.1276465 PMC10641849

[24] Huang B Huang Y Zhai M. Association of sex with cardiovascular outcomes in heart failure patients with obstructive or central sleep apnea. J Am Heart Assoc. 2024;13:e031186. 38410942 10.1161/JAHA.123.031186 PMC10944038

[25] Wang X Tian L Xu Q Pan X Kong L Zhao H. Association between metabolism and low back pain: a cross-sectional study. J Orthop Surg Res. 2025;20:784. 40841650 10.1186/s13018-025-06218-9 PMC12369122

[26] Zha GC Zhu XR Wang L Li HW. Tranexamic acid reduces blood loss in primary total hip arthroplasty performed using the direct anterior approach: a one-center retrospective observational study. J Orthop Traumatol. 2022;23:12. 35254507 10.1186/s10195-022-00638-7 PMC8901851

[27] Minhas AMK Jain V Li M. Family income and cardiovascular disease risk in American adults. Sci Rep. 2023;13:279. 36609674 10.1038/s41598-023-27474-x PMC9822929

[28] Zhang H Tian W Qi G Sun L Wei X. Activated partial thromboplastin time and mortality in coronary artery bypass grafting patients. Dis Markers. 2022;2022:2918654. 36168325 10.1155/2022/2918654 PMC9509521

[29] Unalp-Arida A Ruhl CE. Increasing gallstone disease prevalence and associations with gallbladder and biliary tract mortality in the US. Hepatology. 2023;77:1882–95. 36631004 10.1097/HEP.0000000000000264

[30] Lim J Wirth J Wu K. Obesity, adiposity, and risk of symptomatic gallstone disease according to genetic susceptibility. Clin Gastroenterol Hepatol. 2022;20:e1083–120. 34217876 10.1016/j.cgh.2021.06.044 PMC8720320

[31] Chou TS Lin CL Chen LW. Waist-to-height ratio for the prediction of gallstone disease among different obesity indicators. Obes Sci Pract. 2023;9:30–41. 36789027 10.1002/osp4.650 PMC9913192

[32] Stender S Nordestgaard BG Tybjaerg-Hansen A. Elevated body mass index as a causal risk factor for symptomatic gallstone disease: a Mendelian randomization study. Hepatology. 2013;58:2133–41. 23775818 10.1002/hep.26563

[33] Yuan S Gill D Giovannucci EL Larsson SC. Obesity, Type 2 diabetes, lifestyle factors, and risk of gallstone disease: a mendelian randomization investigation. Clin Gastroenterol Hepatol. 2022;20:e529–37. 33418132 10.1016/j.cgh.2020.12.034

[34] Barahona Ponce C Scherer D Brinster R. Gallstones, Body Mass Index, C-reactive protein, and gallbladder cancer: mendelian randomization analysis of chilean and european genotype data. Hepatology. 2021;73:1783–96. 32893372 10.1002/hep.31537

[35] Radmard AR Merat S Kooraki S. Gallstone disease and obesity: a population-based study on abdominal fat distribution and gender differences. Ann Hepatol. 2015;14:702–9. 26256899

[36] Tazuma S. Gallstone disease: Epidemiology, pathogenesis, and classification of biliary stones (common bile duct and intrahepatic). Best Pract Res Clin Gastroenterol. 2006;20:1075–83. 17127189 10.1016/j.bpg.2006.05.009

[37] Song Y Ma Y Xie FC. Age, gender, geographic and clinical differences for gallstones in China: a nationwide study. Ann Transl Med. 2022;10:735. 35957733 10.21037/atm-21-6186 PMC9358507

[38] Cruz-Monserrate Z Conwell DL Krishna SG. The impact of obesity on gallstone disease, acute pancreatitis, and pancreatic cancer. Gastroenterol Clin North Am. 2016;45:625–37. 27837777 10.1016/j.gtc.2016.07.010

[39] Cortés VA Barrera F Nervi F. Pathophysiological connections between gallstone disease, insulin resistance, and obesity. Obes Rev. 2020;21:e12983. 31814283 10.1111/obr.12983

[40] Nervi F Miquel JF Alvarez M. Gallbladder disease is associated with insulin resistance in a high risk Hispanic population. J Hepatol. 2006;45:299–305. 16516330 10.1016/j.jhep.2006.01.026

[41] Lu XY Shi XJ Hu A. Feeding induces cholesterol biosynthesis via the mTORC1-USP20-HMGCR axis. Nature. 2020;588:479–84. 33177714 10.1038/s41586-020-2928-y

