# Supplementary material for: Linear relationship between percentage-based waist-to-height ratio and the risk of gallstones among US adults: A cross-sectional study from NHANES 2017 to 2020
Source: Medicine (Baltimore). 2026 Jan 9;105(2):e47151. doi: 10.1097/MD.0000000000047151 (PMC12795096; doi:10.1097/MD.0000000000047151)

Supplementary Figure 1. Restricted cubic spline analysis of WHtR and the risk of gallstones in US adults from NHANES 2017-2020 (N = 3,515). Excluding participants with hypertension, diabetes, coronary heart disease, asthma, and cancer.


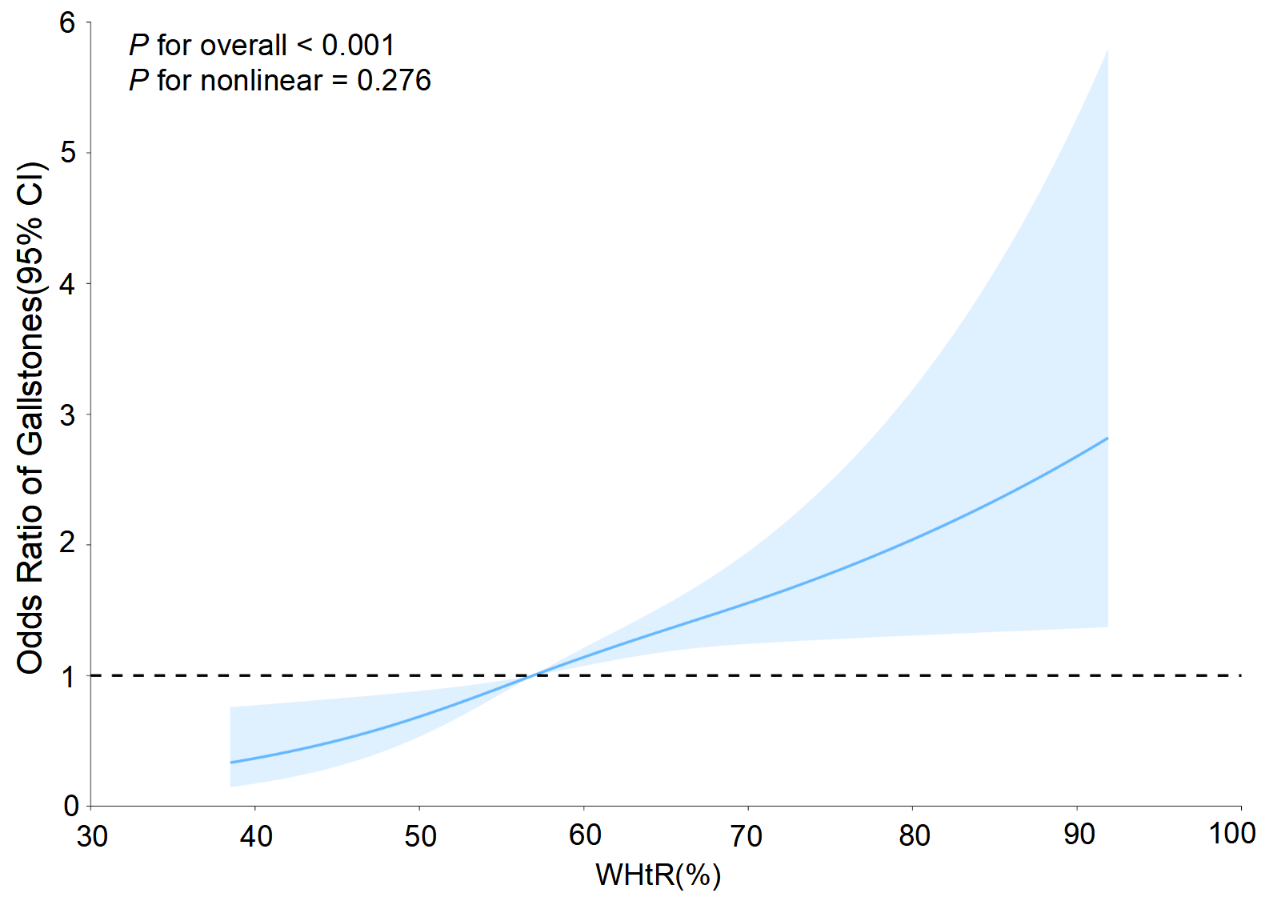

Supplement: Supplementary file 1 [file medi-105-e47151-s001.docx]
